# A Dynamic Clinical Calculator for Estimating Conditional Recurrence-Free Survival After Total Neoadjuvant Therapy for Rectal Cancer and Either Surgery or Watch-and-Wait Management

**DOI:** 10.1001/jamanetworkopen.2022.33859

**Published:** 2022-09-29

**Authors:** Martin R. Weiser, Joanne F. Chou, Jin K. Kim, Maria Widmar, Iris H. Wei, Emmanouil P. Pappou, J. Joshua Smith, Garrett M. Nash, Philip B. Paty, Andrea Cercek, Leonard B. Saltz, Paul B. Romesser, Christopher H. Crane, Julio Garcia-Aguilar, Deborah Schrag, Mithat Gönen

**Affiliations:** 1 Department of Surgery, Memorial Sloan Kettering Cancer Center, New York, New York; 2 Department of Epidemiology and Biostatistics, Memorial Sloan Kettering Cancer Center, New York, New York; 3 Department of Medicine, Memorial Sloan Kettering Cancer Center, New York, New York; 4 Department of Radiation Oncology, Memorial Sloan Kettering Cancer Center, New York, New York

## Abstract

**Importance:**

The risk of recurrence in patients with locally advanced rectal cancer has historically been determined after surgery, relying on pathologic variables. A growing number of patients are being treated without surgery, and their risk of recurrence needs to be calculated differently.

**Objective:**

To develop a dynamic calculator for estimating the probability of recurrence-free survival (RFS) in patients with rectal cancer who undergo total neoadjuvant therapy (TNT) (induction systemic chemotherapy and chemoradiotherapy) and either surgery or watch-and-wait management.

**Design, Setting, and Participants:**

This cohort study included patients who presented with stage II or III rectal cancer between June 1, 2009, and March 1, 2015, at a comprehensive cancer center. Conditional modeling was incorporated into a previously validated clinical calculator to allow the probability of RFS to be updated based on whether the patient remained in watch-and-wait management or underwent delayed surgery. Data were analyzed from November 2021 to March 2022.

**Exposure:**

TNT followed by immediate surgery or watch-and-wait management with the possibility of delayed surgery.

**Main Outcomes and Measures:**

RFS, concordance index, calibration curves.

**Results:**

Of the 302 patients in the cohort, 204 (68%) underwent surgery within 3 months from TNT completion (median [range] age, 51 [22-82] years; 78 [38%] women), 54 (18%) underwent surgery more than 3 months from TNT completion (ie, delayed surgery; median [range] age, 62 [31-87] years; 30 [56%] female), and 44 (14%) remained in watch-and-wait management as of April 21, 2021 (median [range] age, 58 [32-89] years; 16 [36%] women). Among patients who initially opted for watch-and-wait management, migration to surgery due to regrowth or patient choice occurred mostly within the first year following completion of TNT, and RFS did not differ significantly whether surgery was performed 3.0 to 5.9 months (73%; 95% CI, 52%-92%) vs 6.0 to 11.9 months (71%; 95% CI, 51%-99%) vs more than 12.0 months (70%; 95% CI, 49%-100%) from TNT completion (*P* = .70). RFS for patients in the watch-and-wait cohort at 12 months from completion of TNT more closely resembled patients who had undergone surgery and had a pathologic complete response than the watch-and-wait cohort at 3 months from completion of TNT. Accordingly, model performance improved over time, and the concordance index increased from 0.62 (95% CI, 0.53-0.71) at 3 months after TNT to 0.66 (95% CI, 0-0.75) at 12 months.

**Conclusions and Relevance:**

In this cohort study of patients with rectal cancer, the clinical calculator reliably estimated the likelihood of RFS for patients who underwent surgery immediately after TNT, patients who underwent delayed surgery after entering watch-and-wait management, and patients who remained in watch-and-wait management. Delayed surgery following attempted watch-and-wait did not appear to compromise oncologic outcomes. The risk calculator provided conditional survival estimates at any time during surveillance and could help physicians counsel patients with rectal cancer about the consequences of alternative treatment pathways and thereby support informed decisions that incorporate patients’ preferences.

## Key Points

**Question:**

Can a previously validated clinical calculator be adapted to estimate the risk of rectal cancer recurrence dynamically at different time points for patients who defer surgery after complete response to neoadjuvant therapy?

**Findings:**

In this cohort study of 302 patients with stage II or III rectal cancer, recurrence-free survival for patients in the watch-and-wait cohort at 12 months from completion of total neoadjuvant therapy resembled recurrence-free survival in patients who had undergone surgery and had a pathologic complete response. Model performance improved over time, and the concordance index increased from 0.62 at 3 months after total neoadjuvant therapy to 0.66 at 12 months.

**Meaning:**

These findings suggest that this calculator, which helps show how risk of recurrence changes with time, could help patients decide whether to proceed with surgery after neoadjuvant therapy or to safely hold off and could help physicians adjust follow-up intervals optimally over time.

## Teaser

This cohort study develops a dynamic calculator for estimating the probability of recurrence-free survival (RFS) in patients with rectal cancer who have undergone total neoadjuvant therapy and either surgery or watch-and-wait management.

## Introduction

Estimating recurrence-free survival (RFS) is challenging in patients with locally advanced rectal cancer (AJCC [American Joint Committee on Cancer] category T3/4 or N1/2) who undergo total neoadjuvant therapy (TNT) (systemic chemotherapy and chemoradiotherapy) and have the option of watch-and-wait management with the goal of avoiding surgery and preserving the rectum. Long-term outcomes depend on the degree of response and final pathologic stage,^1^ and the likelihood of RFS for a patient undergoing watch-and-wait management can change over time. Currently available models do not allow for dynamic predictions and can provide only a static prediction after resection of the primary tumor.^1,2,3,4,5^

TNT improves delivery of systemic therapy, increases response to treatment, and facilitates nonoperative protocols.^6,7^ The RAPIDO (Rectal Cancer and Preoperative Induction Therapy followed by Dedicated Operation) and PRODIGE 23 (Partenariat de Recherche en Oncologie Digestive) trials recently observed better oncologic outcomes and longer metastasis-free survival in patients with locally advanced rectal cancer who underwent TNT compared with patients who underwent standard chemoradiotherapy.^8,9^ Furthermore, the recently completed OPRA (Organ Preservation in Rectal Adenocarcinoma) trial found that more than 50% of patients with locally advanced rectal cancer treated with TNT can preserve the rectum.^7^

Watch-and-wait management offers the potential to avoid surgery-associated morbidity and bowel, urinary, and sexual dysfunction that can permanently impair the patient’s quality of life.^10^ For a subset of patients with tumors in the low rectum, watch-and-wait offers the potential to avoid a permanent colostomy. The approach is based on the observation that patients with a clinical complete response to neoadjuvant therapy can be monitored and any tumor regrowth can be effectively treated with delayed surgery without compromising oncologic outcome.^11,12,13^ However, patients with clinical complete response, as determined with proctoscopy or magnetic resonance imaging (MRI), may still have residual microscopic disease that will eventually regrow into clinically apparent disease. Retrospective studies indicate that clinical complete response is seen in 30% to 40% of patients who undergo TNT and regrowth occurs in 20% to 30% of those who enter watch-and-wait management. Primary tumor regrowth is most common in the first year after completion of TNT.^12,13^

The commonly used TNM (tumor, node, metastasis) pathologic classification system of the AJCC and the Union for International Cancer Control as well as prognostic models based on tumor regression grade,^1,2^ the neoadjuvant rectal score,^5^ and conventional clinical calculators^3^ are static, rely on pathologic stage, and are inadequate for predicting the likelihood of distant recurrence in patients who undergo watch-and-wait management. The goal of this study was to expand the range of our previously described clinical calculator for estimating the likelihood of recurrence after rectal cancer treatment^4^ by applying the principles of conditional survival to provide dynamic estimations for patients who underwent TNT and then entered watch-and-wait management with the possibility of delayed surgery.

## Methods

This report follows the Strengthening the Reporting of Observational Studies in Epidemiology (STROBE) reporting guidelines for cohort studies. The study was approved by the institutional review board of Memorial Sloan Kettering Cancer Center with a waiver for informed consent because it was a retrospective analysis of previously collected data with no more than minimal risk to participants.

### Patients

We identified patients with locally advanced rectal cancer who were seen in the colorectal surgical oncology clinic of Memorial Sloan Kettering between June 1, 2009, and March 1, 2015, and who received TNT. We defined locally advanced rectal cancer as adenocarcinoma with a distal margin of 15 cm or less from the anal verge on endoscopy, staged with endorectal ultrasound or MRI as clinical AJCC tumor classification (cT) 3 or 4 and clinical nodal classification (cN) 0 or with any cT and cN1 or 2,^14^ in line with the guidelines of the National Comprehensive Cancer Network.^15^ Patients were excluded if they had recurrent or metastatic disease (based on pretreatment computed tomography [CT] of the chest, abdomen, and pelvis), previous surgical treatment for rectal cancer, or concurrent fistulizing inflammatory bowel disease of the rectum.

### TNT Regimens

TNT consisted of induction chemotherapy in the form of mFOLFOX6 (fluorouracil, leucovorin, oxaliplatin) for 8 cycles, CAPOX (capecitabine and oxaliplatin) for 5 cycles, or FLOX (weekly fluorouracil and leucovorin and biweekly oxaliplatin)^16,17^ prior to chemoradiotherapy. Chemoradiotherapy commenced 2 to 3 weeks after completion of induction chemotherapy and typically included 27 to 30 fractions with concurrent infusional fluorouracil at 225 mg/m^2^ or oral capecitabine at 825 mg/m^2^ twice daily. Patients underwent 3-dimensional conformal radiotherapy or intensity-modulated radiotherapy, receiving 45 Gy in 1.8-Gy fractions to the pelvis, with a 9-Gy boost to the tumor. In 3-dimensional conformal radiotherapy, the boost involved 3 additional 1.8-Gy fractions (for a total of 54 Gy in 28 fractions), whereas in intensity-modulated radiotherapy the boost involved dose painting to 2.0 Gy per fraction (for a total of 54 Gy in 27 fractions).^16^

### Assessment of Response to TNT

Within 3 months of completing TNT, patients underwent a repeated evaluation with proctoscopy, CT, and MRI. Surgery was recommended to patients with incomplete response. The possibility that surgery would identify a pathologic complete response^2,18^ was discussed with patients who had a clinical complete response (absence of viable tumor on proctoscopy and MRI).^1,2,4,11,12,16^ Patients electing watch-and-wait management entered a close-observation protocol.^12^ Briefly, observation included proctoscopy every 3 to 4 months and MRI every 3 to 6 months for the first 2 years, followed by proctoscopy every 6 months and MRI every 6 to 12 months for the next 3 years. CT was performed yearly. Delayed surgery was performed in cases of clinical concern for tumor regrowth^11,12,13^ or patient choice, which could be due to anxiety about continuing watch-and-wait management.

For patients who underwent surgery (performed according to the principles of anatomic total mesorectal excision), surveillance followed the guidelines of the National Comprehensive Cancer Network. Detection of recurrence was based on radiographic evidence (with or without biopsy), colonoscopy, and serum level of carcinoembryonic antigen. Local regrowth was not considered a recurrence.^12,13^

### Pretreatment Characteristics and Surgical Findings

Demographic, clinical, and pathologic characteristics as well as follow-up data were retrieved from institutional databases and manually reviewed via the electronic medical record. Pretreatment characteristics included patient age, tumor distance from the anal verge, cT, and cN. Time from the end of radiotherapy to surgery was also retrieved, as were the following postresection tumor characteristics: pathologic AJCC tumor classification (ypT), pathologic AJCC nodal classification (ypN), presence of large- and small-vessel venous invasion and perineural invasion, number of resected lymph nodes, and number of lymph nodes with metastasis (positive lymph nodes). Downstaging was determined by comparing the pretreatment cT category, cN category, and AJCC disease stage as defined in the fifth edition of the AJCC *Cancer Staging Manual* (AJCC 5)^14^ with the postoperative pathologic T category (ypT), N category (ypN), and AJCC disease stage.

### Development of Dynamic Clinical Calculator

**Figure 1.**
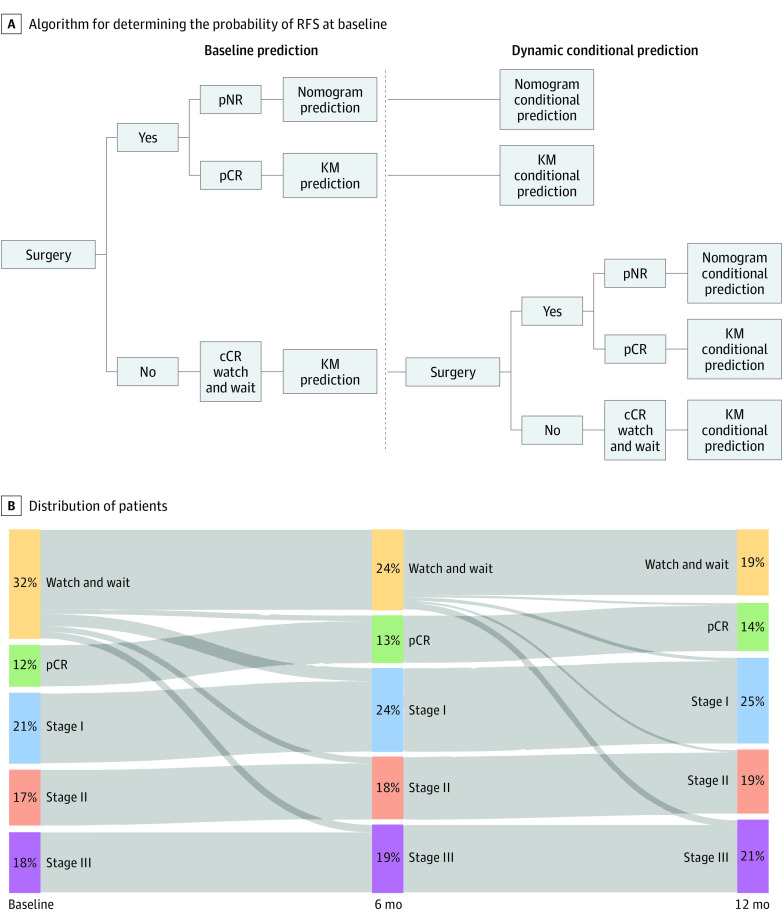
Algorithm and Distribution of Patient Groups Over Time A, Algorithm for estimating the probability of recurrence-free survival (RFS) at baseline (3 months after completion of total neoadjuvant therapy [TNT]) and for updating it at subsequent conditional time points (using the previously validated clinical calculator^4^). B, Distribution of the 302 patients at baseline (3 months), 6 months, and 12 months after completion of TNT. Over time, patients moved from the watch-and-wait cohort due to regrowth or by choice and were recategorized by pathologic stage. cCR indicates clinical complete response; KM, Kaplan-Meier; pCR, pathologic complete response (pathologic classification T0N0); pNR, pathologic incomplete response; stage I, pathologic classification T1 or T2 with N0; stage II, pathologic classification T3 or T4 with N0; and stage III, pathologic classification T1 to T4 with N1 or N2.

We introduced several modifications into our previously validated clinical calculator to provide dynamic estimations in patients treated with watch-and-wait management, immediate surgery, or delayed surgery.^4^ In accordance with the current practice of assessing response 8 to 12 weeks following TNT, RFS was defined for all patients in the study cohort as time from 3 months after the end of TNT (baseline) until the date of recurrence or death. Estimations are provided according to 3 possible clinical scenarios (Figure 1A): resection with pathologic complete response (ypT0N0), resection with pathologic incomplete response, and continued watch-and-wait management with clinical complete response. For patients with pathologic complete response or clinical complete response, the 5-year RFS estimation is based on Kaplan-Meier estimates (eFigure 1 in the Supplement).^4^ For patients with incomplete pathologic response, the estimated likelihood is based on a Cox regression model (nomogram) incorporating the following prognostic factors: AJCC ypT category, number of positive lymph nodes, tumor distance from the anal verge, large- and small-vessel venous invasion, and perineural invasion (eFigure 2 in the Supplement).^4^

Another adaptation was the addition of dynamic landmarking to account for the fact that a recurrence may be detected in follow-up. Estimated 5-year RFS was calculated using dynamic prognostication with conditional survival estimates. An estimation of the probability (*S*) of remaining free of recurrence and alive for *t* months calculated at baseline (3 months after completion of TNT) can be updated with the knowledge that no recurrence was evident at *t_0_* months: *S*(*t*)*/S*(*t_0_*). To examine whether delay of surgery was associated with poor prognosis, we analyzed RFS following rectal resection in relation to whether surgery was performed during 1 of the following intervals after completion of TNT: 3.0 to 5.9 months, 6.0 to 11.9 months, or 12.0 or more months.

### Concordance and Calibration

The general schema for determining the likelihood of remaining free of recurrence for 5 years is shown in Figure 1A. The performance of the clinical calculator was measured for 3 landmark time points: 3 (baseline), 6, and 12 months from completion of TNT. The Uno concordance index was estimated using inverse probability weights.^19^ This index represents the probability that given 2 randomly selected patients, the patient who had a recurrence first had a higher estimated probability of recurrence. Values are interpreted similarly to the area under the receiver operating characteristic curve, with 0.5 corresponding to random chance and 1.0 representing correct estimations for all patients.^20^

In addition to discriminatory performance, the clinical calculator was evaluated with calibration curves by plotting the estimated probabilities of 5-year RFS against the actual RFS rates. If the points fall on or near the 45° line, the model is said to have good calibration, and the projected outcome matches the observed outcome. If the points fall above the 45° line, the model is said to underestimate outcome probabilities. If the points fall below the 45° line, the model is said to overestimate outcome probabilities.

### Statistical Analysis

Differences in RFS between groups were compared using the log-rank test. All statistical analyses were performed with R version 3.6.0 (R Foundation for Statistical Computing, Vienna, Austria). All *P* values were two-sided, and *P* < .05 was considered indicative of a significant difference.

## Results

Of 313 patients who underwent TNT during the study period, 9 patients with clinical evidence of disease who refused surgery and 2 patients with distant metastasis identified within 3 months of TNT completion were excluded. The remaining 302 patients constituted the study cohort: 204 patients who underwent surgery within 3 months of TNT completion (median [range] age, 51 [22-82] years; 78 [38%] women), and 98 patients who entered watch-and-wait management, with 54 undergoing surgery more than 3 months after TNT completion (median [range] age, 62 [31-87] years; 30 [56%] women) and 44 who remained in watch-and-wait management as of April 21, 2021 (median [range] age, 58 [32-89] years; 16 [36%] women). Median follow-up for patients in the watch-and-wait group was 52 months (range, 2-103 months), and 75 of the 302 patients had a recurrence or died. The clinicopathologic characteristics of patients who underwent surgery 3 or fewer months after TNT (n = 204), patients who underwent surgery more than 3 months after TNT (n = 54), and patients remaining in watch-and-wait management are listed in the Table.^21^ Migration of patients from watch-and-wait management to surgery and associated pathologic stage are shown in Figure 1B.

**Table.** Patient and Disease Characteristics

Characteristic	Patients, No. (%)		
	Immediate surgery (n = 204)	Watch-and-wait management	
		Delayed surgery (n = 54)	No surgery (n = 44)^a^
Age, median (range), y	51 (22-82)	62 (31-87)	58 (32-89)
Sex			
Female	78 (38)	30 (56)	16 (36)
Male	126 (62)	24 (44)	28 (64)
DTAV ≤5 cm	46 (23)	26 (48)	19 (43)
Months to surgery or last evaluation, median (range)^b^	1.9 (0.3-3.0)	6.3 (3.0-46.2)	51.2 (18.3-79.3)
cT category			
1	1 (1)	1 (2)	0
2	10 (5)	4 (7)	4 (9)
3	168 (82)	39 (72)	39 (89)
4	25 (12)	10 (19)	1 (2)
cN category			
0	18 (9)	8 (15)	18 (41)
1	158 (77)	40 (74)	25 (57)
2	28 (14)	6 (11)	1 (2)
Pretreatment clinical stage according to AJCC 5			
II	18 (9)	8 (15)	18 (41)
III	186 (91)	46 (85)	26 (59)
Pathologic tumor response			
Complete	37 (18)	7 (13)	NA
Incomplete	167 (82)	43 (80)	NA
Unknown	0	4 (7)	NA
ypT category			
0	40 (20)	9 (17)	NA
1	17 (8)	3 (6)	NA
2	56 (27)	21 (39)	NA
3	86 (42)	13 (24)	NA
4	5 (3)	5 (9)	NA
Unknown	0	3 (6)	NA
ypN category			
0	150 (74)	39 (72)	NA
1/2	54 (26)	11 (20)	NA
Unknown	0	4 (7)	NA
Pathologic stage according to AJCC 8			
0	37 (18)	7 (13)	NA
I	63 (31)	21 (39)	NA
IIa	46 (23)	10 (19)	NA
IIb	4 (2)	1 (2)	NA
IIIa	11 (5)	4 (7)	NA
IIIb	37 (18)	7 (13)	NA
IIIc	6 (3)	0	NA
Unknown	0	4 (7)	NA
Venous invasion^c^			
Absent	170 (83)	39 (72)	NA
Present	34 (17)	12 (22)	NA
Unknown	0	3 (6)	NA
PNI			
Absent	165 (81)	35 (65)	NA
Present	39 (19)	16 (30)	NA
Unknown	0	3 (6)	NA
Change in AJCC 5 stage^d^			
Downstaged	142 (70)	37 (69)	NA
No change	60 (29)	12 (22)	NA
Upstaged	2 (1)	1 (2)	NA
Missing data	0	4 (7)	NA

Abbreviations: AJCC 5, AJCC *Cancer Staging Manual*, fifth edition; AJCC 8, AJCC *Cancer Staging Manual*, eighth edition; cN, clinical nodal classification; cT, clinical tumor classification; DTAV, distance of tumor from the anal verge; NA, not applicable; PNI, perineural invasion; ypN, pathologic nodal classification; ypT, pathologic tumor classification.

^a^ As of data lock on April 21, 2021.

^b^ From completion of total neoadjuvant therapy.

^c^ Small-vessel lymphatic or venous invasion or large-vessel intramural or extramural venous invasion.

^d^ Upstaged indicates postoperative pathologic category or stage higher than the pretreatment clinical category or stage; downstaged, postoperative pathologic category or stage lower than the pretreatment clinical category or stage.

**Figure 2.**
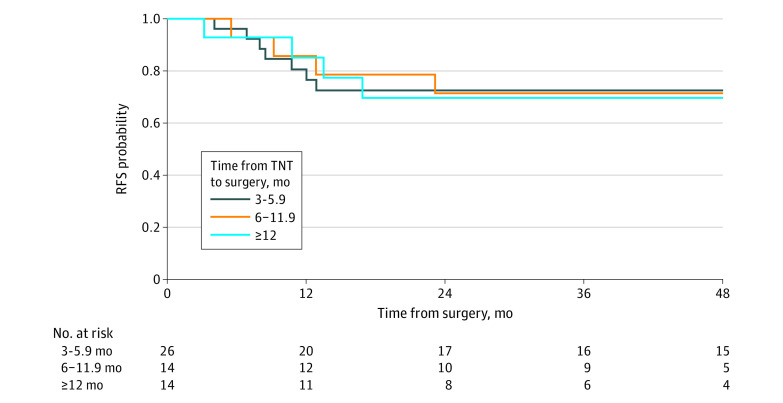
Recurrence-Free Survival (RFS) Relative to Surgery Delay Interval Kaplan-Meier RFS curves for patients who underwent surgery 3.0 to 5.9 months, 6.0 to 11.9 months, or 12.0 or more months following completion of total neoadjuvant therapy (TNT).

Of the 54 patients in the watch-and-wait cohort who eventually underwent surgery for regrowth or by choice, 26 underwent surgery 3.0 to 6.9 months after completion of TNT, 14 underwent surgery 6.0 to 11.9 months after completion of TNT, and 14 underwent surgery 12.0 or more months after completion of TNT. Three-year RFS did not differ significantly between these groups: 73% (95% CI, 57%-92%), 71% (95% CI, 51%-99%), and 70% (95% CI, 49%-100%), respectively (*P* = .70) (Figure 2).

**Figure 3.**
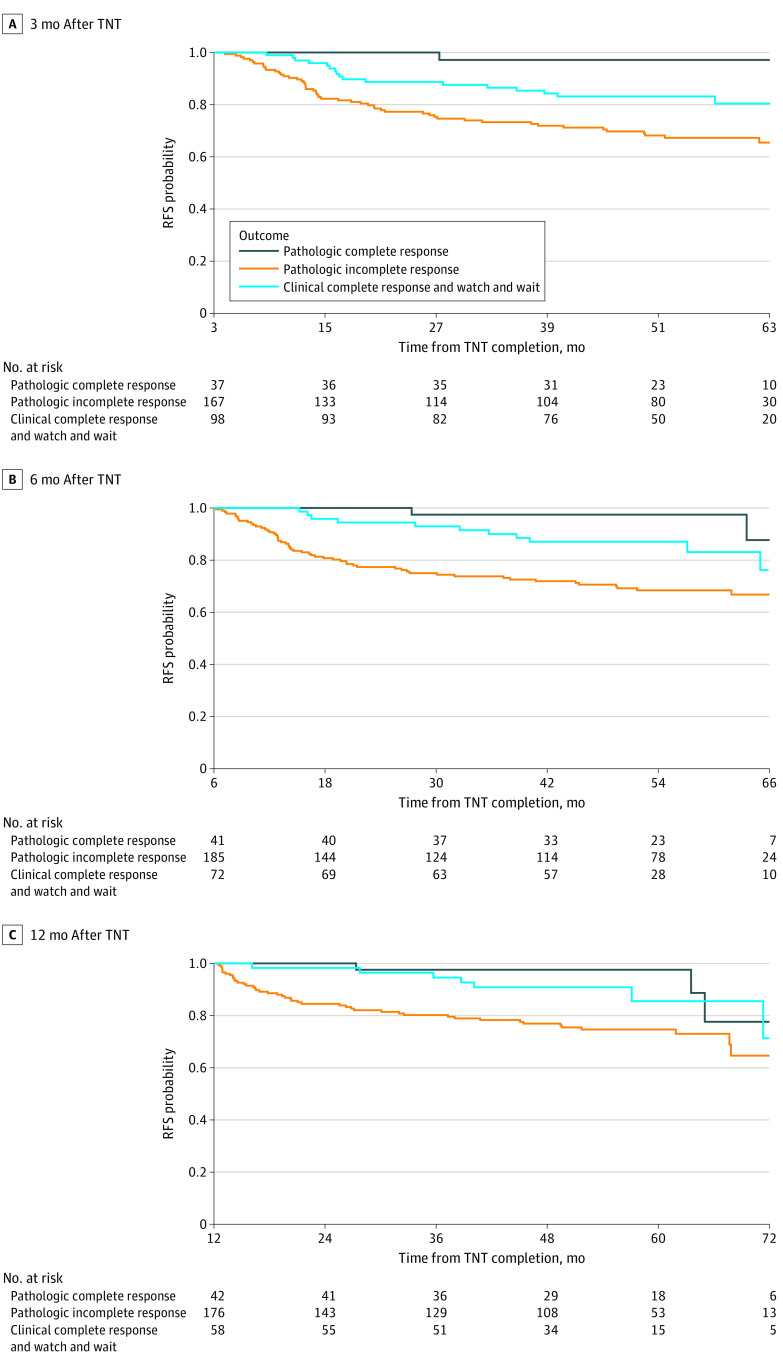
Recurrence-Free Survival (RFS) From 3, 6, and 12 Months After Total Neoadjuvant Therapy (TNT) Probabilities of RFS starting from 3 (A), 6 (B), and 12 (C) months after completion of TNT for patients who underwent surgery and had a pathologic complete response, patients who underwent surgery and had a pathologic incomplete response, and patients who had a clinical complete response and remained in watch-and-wait management.

**Figure 4.**
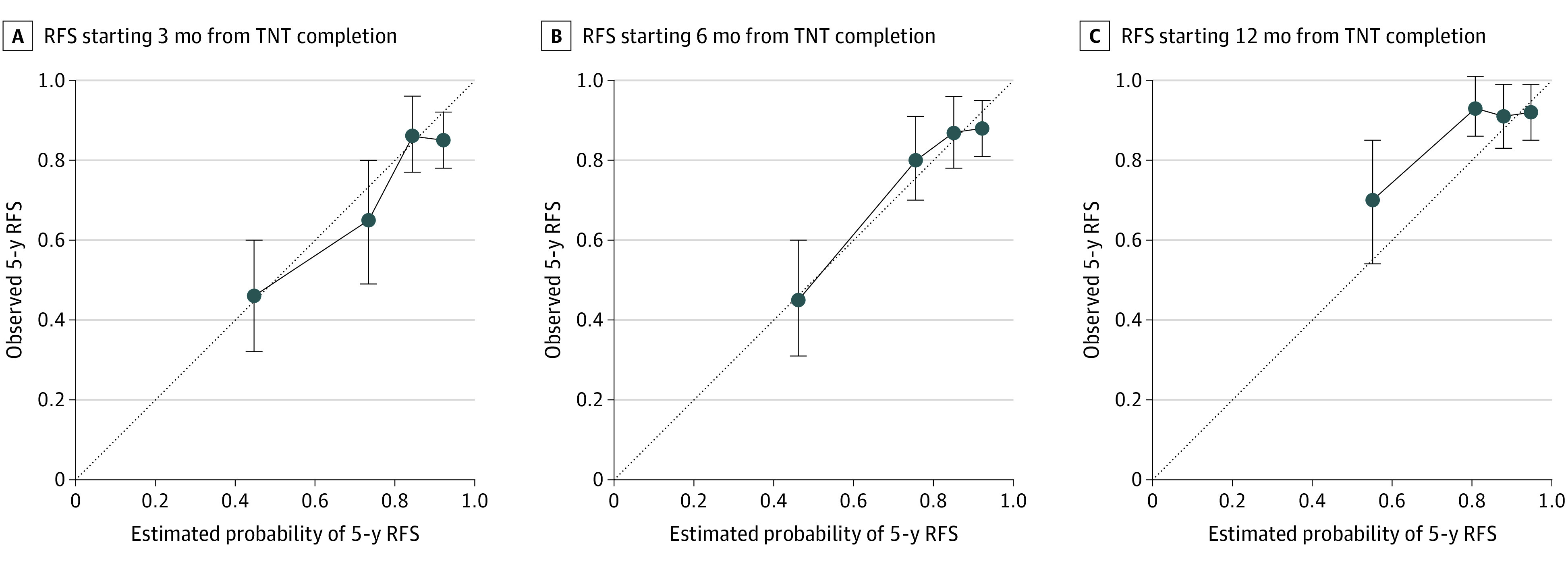
Calibration Curves Calibration curves for calculating the probabilities of 5-year recurrence-free survival (RFS) starting from 3 (A), 6 (B), and 12 (C) months after completion of total neoadjuvant therapy (TNT). The 45° diagonal and quartiles with 95% CIs are indicated.

Five-year RFS curves originating at 3, 6, and 12 months after completion of TNT are shown in Figure 3 for patients who underwent surgery and had a pathologic complete response to TNT, patients who underwent surgery and had a pathologic incomplete response to TNT, and patients who had a clinical complete response to TNT and remained in watch-and-wait management. RFS in the watch-and-wait group closely approximated RFS in the pathologic complete response group, with an associated increase in Uno concordance index over time: 0.62 (95% CI, 0.53-0.71), 0.64 (95% CI, 0.52-0.74), and 0.66 (95% CI, 0-0.75) for RFS curves originating at 3, 6, and 12 months, respectively. The calibration plots for estimating the probability of 5-year RFS starting from 3, 6, and 12 months after TNT completion approximated the 45° diagonal (Figure 4). However, for prognosis at 12 months after TNT completion, the model appeared to underestimate the likelihood of 5-year RFS for some patients (Figure 4C).

## Discussion

For patients with locally advanced rectal cancer who underwent TNT followed by immediate surgery or watch-and-wait management, applying dynamic landmarking and conditional survival to a previously validated clinical calculator^4^ gave us the ability to recalculate the probability of RFS at different points during posttreatment surveillance. Patients who opt for watch-and-wait include those who have a true complete response and those who have microscopic residual disease that will become clinically apparent over time (local regrowth) and will require surgery. For patients with a clinical complete response sustained over time, our data indicate that the likelihood of remaining free of recurrence approximates that of patients with a pathologic complete response after undergoing surgery. Not surprisingly, the accuracy of estimating the likelihood of RFS increases with time since completion of TNT. The increase from a concordance index of 0.62 at 3 months to 0.66 at 12 months is greater than the typical difference in concordance index between successive iterations of AJCC staging.^22^

In patients who underwent delayed surgery, RFS following rectal resection was not statistically different based on the time from completion of TNT to surgery. This finding indicates that oncologic outcome was not compromised by the delay. It also suggests that local tumor regrowth does not lead to metastases. Propensity to metastases is likely related to the biology of the primary tumor at diagnosis. Tumor phylogenetic studies may be able to further test this hypothesis.^23^ Fernandez et al^24^ also reported that more than 88% of regrowth occurs within the first year after neoadjuvant therapy and that the risk of distant recurrence in patients with sustained complete response is low.

### Limitations and Strengths

This study has limitations. It was subject to the selection bias inherent in retrospective analyses, which we minimized by closely following the methodological criteria established by the AJCC Precision Medicine Core.^25^ The study’s strengths include standardized surveillance and resection procedures, comprehensive histologic assessment by specialized pathologists, and the availability of granular clinical and demographic data with long follow-up. As has been done with other calculators, updates and modifications can be performed over time as new data sets become available.^26,27,28^

The calculator is unique and dynamic, able to provide risk estimation at any point during surveillance following modern rectal cancer treatment in which patients may choose observation, immediate surgery, or delayed surgery. Other outcome models, including those that rely on AJCC, tumor regression grade,^1,2^ the neoadjuvant rectal score,^5^ and conventional clinical calculators,^3^ require anatomic staging factors, can only be calculated immediately after surgery, and cannot incorporate watch-and-wait management. Furthermore, the addition of conditional survival allows refinement of recurrence estimation based on being free of disease over time. This allows physicians to answer the commonly asked question during follow-up: what is the chance of my tumor recurring now?

## Conclusions

A web interface^29^ will allow patients and physicians to input the date of TNT completion, the date of surgery (if performed), and the date of last follow-up to obtain a conditional estimate of the probability of remaining free of recurrence for 5 years. Being able to obtain a more accurate probability estimate as new data becomes available over time will aid patients and physicians in making appropriate adjustments to continued follow-up.

The calculator can also be used at decision points along the course of rectal cancer treatment as patients and physicians consider watch-and-wait management vs surgery. In this way, the calculator supports clinical decision-making that incorporates patients’ preferences and enables them to have clear expectations about likely outcomes.

## Notes

Supplement. **eFigure 1.** Kaplan-Meier 5-Year RFS Curve for Patients With Pathologic Complete Response or Clinical Complete Response to Neoadjuvant Therapy **eFigure 2.** Nomogram for Estimating RFS in Patients With Incomplete Pathologic Response to Neoadjuvant Therapy
