# Supplementary material for: A Dynamic Clinical Calculator for Estimating Conditional Recurrence-Free Survival After Total Neoadjuvant Therapy for Rectal Cancer and Either Surgery or Watch-and-Wait Management
Source: JAMA Netw Open. 2022 Sep 29;5(9):e2233859. doi: 10.1001/jamanetworkopen.2022.33859 (PMC9523500; doi:10.1001/jamanetworkopen.2022.33859)
Supplement: Supplement. — eFigure 1. Kaplan-Meier 5-Year RFS Curve for Patients With Pathologic Complete Response or Clinical Complete Response to Neoadjuvant Therapy eFigure 2. Nomogram for Estimating RFS in Patients With Incomplete Pathologic Response to Neoadjuvant Therapy [file jamanetwopen-e2233859-s001.pdf]

Supplemental Online Content

Weiser MR, Chou JF, Kim JK, et al. A dynamic clinical calculator for estimating conditional recurrence-free survival after total neoadjuvant therapy for rectal cancer and either surgery or watch-and-wait management. *JAMA Netw Open.* 2022;5(9):e2233859. doi:10.1001/jamanetworkopen.2022.33859

eFigure 1. Kaplan-Meier 5-Year RFS Curve for Patients With Pathologic Complete Response or Clinical Complete Response to Neoadjuvant Therapy

eFigure 2. Nomogram for Estimating RFS in Patients With Incomplete Pathologic Response to Neoadjuvant Therapy

This supplemental material has been provided by the authors to give readers additional information about their work.

© 2022 Weiser MR et al. *JAMA Network Open.*

eFigure 1. Kaplan-Meier 5-Year RFS Curve for Patients With Pathologic Complete Response or Clinical Complete Response to Neoadjuvant Therapy.[4]

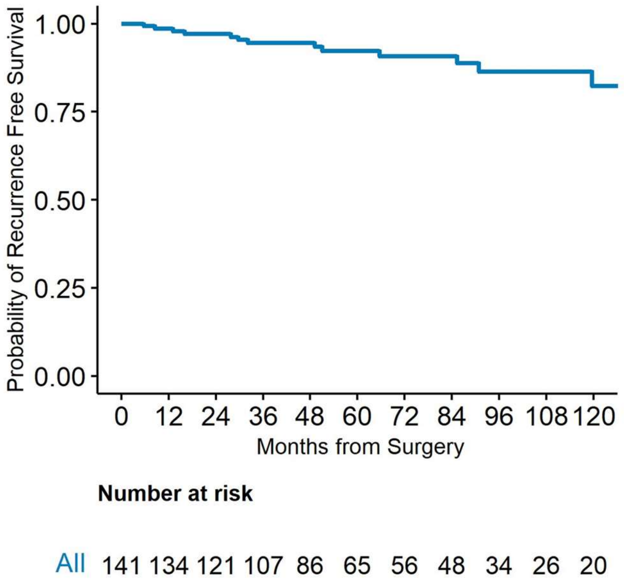

© 2022 Weiser MR et al. *JAMA Network Open.*

eFigure 2. Nomogram for Estimating RFS in Patients With Incomplete Pathologic Response to Neoadjuvant Therapy.[4]

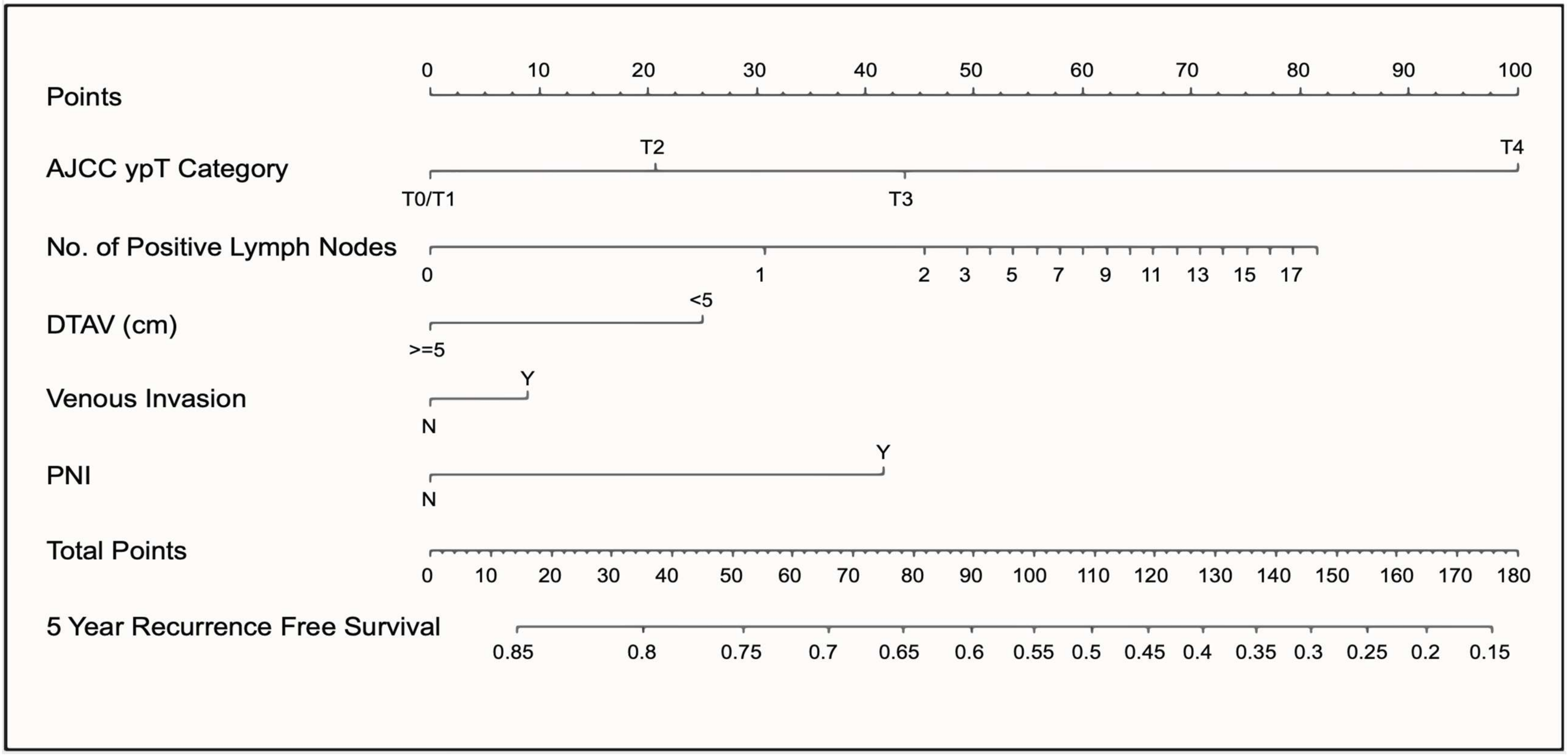

For each prognostic variable, draw a straight line up to the Points axis to determine the points for that variable. Repeat this process for each variable. Add the points for all the variables and locate the sum on the Total Points axis. Draw a straight line from Total Points down to 5-year recurrence-free survival. Venous invasion is small-vessel lymphatic or venous invasion or large-vessel intramural or extramural venous invasion. DTAV, distance of tumor from the anal verge; PNI, perineural invasion.

© 2022 Weiser MR et al. *JAMA Network Open.*
